# Clinical Outcomes of Prostatic Artery Embolization in Patients with Benign Prostatic Hyperplasia: A Prospective Clinical Study

**DOI:** 10.5152/tud.2022.22004

**Published:** 2022-05-01

**Authors:** Kazım Dogan, Ahmet Erbagci, Haluk Sen, Selim Kervancioglu, Mehmet Sakıp Erturhan, İlker Seckiner, Ömer Bayrak

**Affiliations:** 1 Department of Urology, LIV Hospital, Gaziantep Turkey; 2 Department of Urology, Gaziantep University Faculty of Medicine, Gaziantep, Turkey; 3 Department of Urology, Sanko University Faculty of Medicine, Gaziantep, Turkey

**Keywords:** Prostatic hyperplasia, prostatic artery embolization, geriatrics

## Abstract

**Objective:**

To determine the clinical outcomes of prostatic artery embolization applied to patients with benign prostatic hyperplasia.

**Material and methods:**

The study includes 30 patients diagnosed with benign prostatic hyperplasia in the urology clinic between 2012 and 2016, for whom anesthesia was contraindicated due to advanced age and comorbidities and who underwent prostatic artery embolization. These patients were evaluated before the procedure and in the 1st, 3rd, 6th, and 12th months after the procedure.

**Results:**

The mean prostate volume of the patients was 68 cm³ before the procedure and 45 cm³ 12 months after the procedure. A statistically significant decrease was observed (*P* = .001). The mean prostate-specific antigen value was 4.9 ng/dL before the procedure and 2.8 ng/dL 12 months after the procedure (*P* = .008). The mean *Q*_max_ value was 0 mL/s before the procedure and 12 mL/s 12 months after the procedure (*P* = .001). The mean international prostatic symptom scores value was 35 before and 16 twelve months after the procedure (*P* = .001). While the international index of erectile function value was 8.25 before the procedure, it was 8.46 12 months after the procedure (*P* = .32). The quality of life index value was measured as 3.02 before the procedure and 3.09 twelve months after the procedure; a statistically significant difference was determined (*P* = .027).

**Conclusion:**

Prostatic artery embolization, which is a minimally invasive procedure, can be applied as a safe and effective method to patients with benign prostatic hyperplasia who cannot tolerate anesthesia due to advanced age and comorbidities.

**Main Points**

- Benign prostatic hyperplasia is a common health problem in men aged 50 and over.
- There are medical and surgical options for the treatment of benign prostatic hyperplasia.
- The gold standard treatment method is transurethral resection of the prostate.
- Prostatic artery embolization method can be used effectively and safely in patients who cannot receive anesthesia due to comorbidity.

## Introduction

Benign prostatic hyperplasia (BPH) is defined as an increased number of epithelial and stromal cells in the periurethral areas of the prostate gland. Benign prostatic hyperplasia is a very common health problem in the male population, especially over age 50, and its incidence increases with age, reaching 88% in the 80s and almost 100% in the 90s.^1^

Many irritative symptoms are seen in BPH, especially related to bladder dysfunction.^2^ Quality of life decreases due to some sexual dysfunctions and symptoms that include difficulty in starting to void and dripping after voiding, sudden urge to urinate, frequent urination, dysuria and nocturia, and feelings of insufficient ejaculation.^2-4^

Benign prostatic hyperplasia-associated lower urinary tract symptoms (LUTS) present discomfort for the patient, but there are many medical and surgical treatments available. Alpha-blockers and other drugs used in the medical treatment of BPH have not been found to not have any effect and in long-term use have side effects, including orthostatic hypotension, erectile dysfunction, and decreased libido.^4,5^ Therefore, various surgical methods such as transurethral resection of the prostate, open prostatectomy, and transurethral incision of the prostate are applied instead of medical treatment in these patients.^6,7^ However, other alternative methods are sought for elderly patients who cannot undergo anesthesia and surgery due to diabetes mellitus, hypertension, coronary artery disease, and other problems. Prostatic stent application, prostatic sling application, alcohol incision application to the prostate, local laser application on the prostate, and intermittent catheter exchange application are among the main methods applied to these patients.^8,9^

Prostatic artery embolization (PAE) is a relatively a new method. It was first performed in 1977 on a patient with prostate cancer^10^ because of the presence of hematuria. In recent years, Wilisch et al^11^ have shown that PAE can be effective in patients with LTUS due to BPH. In 2000, DeMeritt et al^12^ tried to explain the mechanism of action of PAE as widespread devascularization of the enlarged prostate gland.^13^ However, it is not known precisely how PAE corrects AUSS.^14^

Our study aimed to evaluate the efficacy of PAE in patients with BPH for whom anesthesia is risky due to advanced age and additional disease.

## Material and Methods

This research was conducted in our urology clinic between 2012 and 2016 as a prospective experimental study.

A total of 30 patients with the diagnosis of BPH in the context of advanced age and comorbidity were included in this study when it was determined they met the inclusion criteria. After meeting patients who met the inclusion criteria of the study, the purpose of the study was explained and informed consent was received from those who agreed to participate in the study.

Our study looked for patients with advanced age and comorbidity (Charlson comorbidity index ≥2) who demonstrated maximum flow rate (*Q*_max_) of less than 12 mL/s according to uroflowmetry and demonstrated normal to high prostate-specific antigen (PSA) value for their age; we then accepted patients with benign12-quadrant biopsy results seen by transrectal ultrasonography (TRUS) under local anesthesia; prostate volumes (PV) calculated as greater than 40 cm^3^ by transrectal USG; and international prostatic symptom scores (IPSS) greater than 12.

Our study excluded those with a history of urethral stricture in their anamneses, malignant prostate biopsy results, creatinine values greater than 1.4 ng/dL, prostate volumes less than 40 cm³, neurogenic bladder disease, atonic bladder disease, a disease resulting in bladder dysfunction, and/or severe atherosclerosis.

### Procedure

Eligible patients were hospitalized the day before the procedure and complete blood count, routine biochemical tests, and PT INR, APTT tests were performed. Prior to the procedure, the patients received isotonic sodium chloride and 1 g ceftriaxone by intravenous injection. A unilateralurethral catheter was applied to all patients before the procedure. Oral antibiotherapy (levofloxacin) was given for 1 week after the procedure and the urethral catheters were removed in the first week after the procedure.

**Figure 1. a,b.**
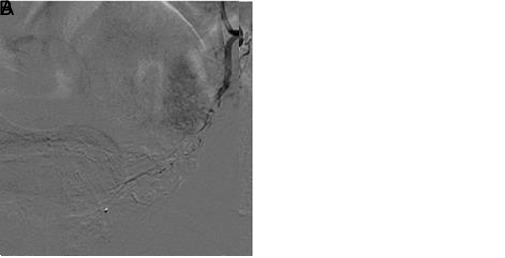
(a) Axial T1-weighted angiography images show pre-procedural. (b) Coronal T1-weighted angiography images show post-procedure.

The interventional radiologist injected the femoral artery of the patient with 5-F sheath (Radifocus, Terumo, Japan) under local anesthesia. Radiopaque contrast media (Optiray 320 Mallinckrodt-Turkey) was administered via 5-F hydrophilic catheter (Terumo, Radifocus guide wire, Japan) and advanced to the hypogastric artery with the help of angiographic imaging (Figure 1a). Then, microcatheters (multiple manufacturers) between 2.0 F and 2.8 F were placed in the selective prostatic artery and 200 µg nitroglycerin was injected to prevent vasospasm. Embolization was applied by injecting microparticles of 100-300 µm or 300-500 µm poly (vinyl alcohol) structure based on the vessel diameter. Angiographic imaging was repeated and embolization was observed as complete (Figure 1b). Unilateral embolization was applied to all patients.

**Figure 2. a,b.**
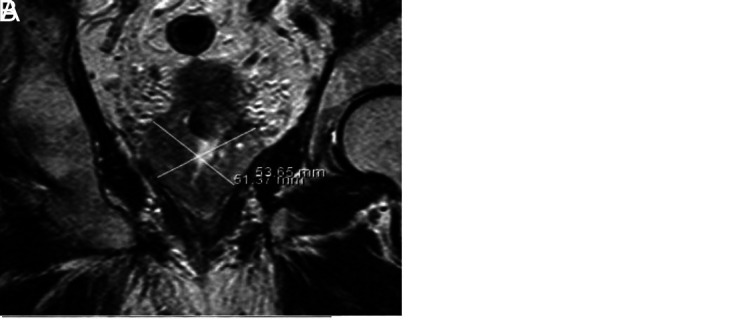
(a) Axial T1-weighted MR images show pre-procedure. (b) Coronal T1-weighted MR images show post-procedure.

Prostate volumes of the patients were calculated by pelvic magnetic resonance (MR) (Figure 2a). Patients were called for check-up in the 1st, 3rd, 6th, and 12th months after the procedure. Their PV with pelvic MR (Figure 2b) and PSA, *Q*_max_, IPSS, international index of erectile function (IIEF), quality of life index (Qol), and creatinine values were measured and the results compared.

### Statistical Analysis

After the study data was coded by the researcher, the Statistical Package for Social Sciences (SPSS) for Windows 22.0 (IBM SPSS Corp.; Armonk, NY, USA) was applied. Compliance of numerical data with normal distribution was tested with the Shapiro–Wilk test. Friedman 2-way analysis of variance was used to compare non-normally distributed variables at different times. In comparisons, CI was taken as 95% and *P* < .05 was accepted as significant.

### Ethical Approval

This research was conducted in accordance with the provisions of the Declaration of Helsinki, which was revised in Brazil in 2013. The Gaziantep University Clinical Research Ethics Committee (April 20, 2014-subject 176) gave permission to conduct the study. In addition, written and verbal permissions were obtained from the participants, who stated they understood that participation in the research was completely voluntary and that they could withdraw from the study whenever they wanted.

## Results

The study included 30 patients; of these, 28 patients with inserted catheters were followed, as they could not urinate before the procedure; the remaining 2 patients were able to urinate. The mean age of the 30 patients in our study was 79.3 ± 6.8 years. The mean body mass index was 25.3 ± 4.3, and the mean Charlson comorbidity index was 3.0 ± 1.8. Prostate volume was 68.0 ± 23.25 cm^3^, PSA was 4.9 ± 1.2 ng/mL, Q_max _was 0 mL/sec, IPSS score was 35 ± 3.0, IEFF was 8.25 ± 0.67, and Qol score was 3.2 ± 0.28 (Table 1). 

The mean PV of the patients before the procedure was 68 ± 23.25 cm^3^. After the procedure, it was measured at 61.5 ± 25.5 cm³ in the 1st month, 48.2 ± 15.7 cm³ in the 3rd month, 45.5 ± 16.2 cm³ in the 6th month, and 45.0 ± 16.0 cm³ in the 12th month, representing a statistically significant decrease (*P* = .001). However, when the results of the 6th and 12th months were compared, no statistically significant difference was found (*P* = .14).

The mean PSA level of the patients before the procedure was 4.9 ± 1.2 ng/dL. After the procedure, the level was determined as 3.8 ± 1.25 ng/dL in the 1st month, 3.5 ± 1.1 ng/dL in the 3rd month, 2.85 ± 1.05 ng/dL in the 6th month, and 2.8 ± 1.2 ng/dL in the 12th month. When the mean PSA value at the end of the 12th month was compared to the pre-procedural value of the patients, a statistically significant decrease was determined (*P* = .008). However, when the results of the 6th and 12th months were compared, no statistically significant difference was found (*P* = .30).

While the Q_max _value of 2 patients was determined as 10 ± 1.8 mL/s before the procedure, none of the remaining 28 patients could urinate and all were given permanent urethral tubes. Since uroflowmetry could not be performed on these patients, the *Q*_max _value was accepted as 0 mL/sec. The mean Q_max _value, which was accepted as 0 mL/s before the procedure, was 12 ± 2.0 mL/s in the 1st month, 14 ± 2.5 mL/s in the 3rd month, 14 ± 2.0 mL/s in the 6th month, and 12.0 ± 3.0 mL/s in the 12th month after the procedure, and a statistically significant increase was found (*P* = .001). However, between the results of the 3rd month and the 12th month after the procedure no statistically significant difference was found (*P* = .20).

In the IPSS evaluation of the patients in the outpatient clinic, the mean IPSS value was found to be 35.0 ± 3.0 points. While the mean IPSS value was 22.5 ± 1.5 in the 1st month after the procedure, it was 18.0 ± 2.0 in the 3rd month, 16.0 ± 2.0 in the 6th month, and 16.0 ± 2.0 in the 12th month, and a statistical decrease was detected in the specified months (*P* = .001). After the procedure, however, no statistically significant difference was found between the results of the 3rd and 6th months (*P* = .16) and the 6th and 12th months (*P* = .93).

The IEFF value was 8.25 ± 0.67 points before the procedure. It increased to 8.31 ± 0.6 points in the 1st month, 8.39 ± 0.62 points in the 3rd month, 8.42 ± 0.65 points in the 6th month, and 8.46 ± 0.70 points in the 12th month after the procedure. However, there was no statistically significant difference (*P* = .32).

The Qol value was 3.02 ± 0.28 before the procedure. It was 3.06 ± 0.26 in the 1st month after the procedure, 4.06 ± 0.27 in the 3rd month, 4.96 ± 0.29 in the 6th month, 5.09 ± 0.29 in the 12th month after the procedure. A statistically significant difference occurred in the Qol value (*P* = .027) (Table 2).

At the end of the first week after the procedure, urethral tubes of patients who had been unable to urinate were removed. Twenty-two patients (78.5%) could urinate comfortably, while 6 (21.5%) patients could not urinate. Two patients initially able to urinate were not included in this ratio. Since these patients could not initially urinate, their ability to urinate after the procedure was evaluated as a success criterion and the success rate of this procedure was found to be 78.5%. A prostatic stent was placed in one of these 6 patients; the remaining 5 patients were followed, as they had permanent catheters. No major or minor complications were detected in any of the patients.

## Discussion

The literature was reviewed and discussed in light of the study findings, This study was conducted with elderly patients and patients with high comorbidity averages. The study by Kurbatov et al^15^ determined that the prostate volume decreased from 129.1 cm³ to 71.2 cm³ at the end of the 12th month, but when the 6th month (69.4 cm³) and 12th month (71.3 cm³) values were compared, no statistically significant difference was found. Studies examining the effect of prostatic artery embolization applied to patients with benign prostatic hyperplasia determined patient prostate volumes to decrease after the procedure compared to before.^4,16-23^ This study measured patient prostate volume as averaging 68 ± 23.25 cm³ before the procedure; and measured prostate volume after the procedure as 61.5 ± 25.5 cm³ in the 1st month, 48.2 ± 15.7 cm³ in the 3rd month, 45.5 ± 16.2 cm³ in the 6th month, and 45.0 ± 16.0 cm³ in the 12th month. The study determined patient prostate volumes to decrease after the procedure compared to before, but there was no statistically significant difference comparing the 6th and the 12th month results. The fact that no significant difference was found in values at the end of the 6th and 12th months suggests that the shrinkage in the prostate was completed in the 6th month and did not continue after that. This result is similar to those of previous studies in the literature.^4,15-23^

In 2014, Kurbatov et al^15^ found the PSA value to decrease after the procedure, but there was no statistically significant difference when the post-procedural 3rd, 6th, and 12th month values were compared. The 2017 study by Carnevale et al^17^ found the PSA value to decrease 12 months after the procedure, with no significant difference in results the 3rd and 12th month after the procedure. Our study found a lower mean PSA value in the 12th month after the procedure, but there was no statistically significant difference when the 6th and 12th month results were compared. This suggests that PSA production is decreased due to atrophy of prostate cells. The fact that there is no difference in the PSA value between the 6th and 12th months after the procedure can be explained by the completion of atrophy in the prostate cells in the 6th month. The results of similar studies support our findings. However, there is also a study in the literature in which PAE application did not affect the PSA value^22^; the results of this research do not correlate with those of our study.

Studies conducted to determine the effect of prostatic artery embolization on patients with benign prostatic hyperplasia have determined that the *Q*_max_ values of the patients increased after the procedure compared to before the procedure.^18,21,24,25^ Our study’s findings support these results. Our study found that patient *Q*_max_ values increased after the PAE procedure compared to before, but determined no significant difference between the 3rd and 12th month results. This suggests that the *Q*_max_ value increases due to the reduction of prostate volume resulting from the atrophy of prostate cells and decrease in bladder outlet obstruction. The data we obtained show that the *Q*_max_ value of the patients increased until the end of the 3rd month after the procedure, then continued without significant change until the end of the 12th month. This may be because the atrophy in the prostate continued until the 6th month, but did not cause a clinically significant difference in the *Q*_max_ value after the 3rd month.

International studies have found that the PAE procedure reduces the IPSS score.^17,18,24,25^ The 2018 study by Wang et al^21^ found that the IPSS score decreased after the procedure, but saw no significant difference in the 12th and 24th month results after the procedure. There are also studies in which PAE application did not affect the IPSS value.^22,26^ The results of previous studies are similar to those of our study. The IPSS scores of our patients decreased after the PAE procedure compared before, but we found no significant difference in results in the 3rd, 6th, and 12th month after the procedure. This shows us that IPSS scores decrease due to the reduction of prostate volume resulting from the atrophy of prostate cells and the reduction of bladder outlet obstruction. Considering the IPSS score, we observed that the clinical improvement of the patients gradually decreased in the first 3 months, but there was no significant difference afterwards. This may be because the atrophy in the prostate continued until the 6th month but did not cause a clinically significant difference in the IPSS score after the 3rd month.

The 2014 study by Bagla et al^4^ found no statistically significant difference in the pre-procedure and postoperative 1st month, 3rd month, and 6th month IIEF scores. This study’s findings are similar to the results of our study.^4^ In our study, the IIEF score was 8.25 points before the procedure. After the procedure, the 1st month score increased to 8.31, the 3rd month score increased to 8.39, the 6th month score increased to 8.42, and the 12th month score increased to 8.46; nonetheless, we found no statistically significant difference. We think that although the catheters of the patients were removed after the procedure, IIEF scores were significantly unchanged due to advanced patient age and the presence of chronic diseases.

The 2014 study by Bagla et al^4^ found the Qol score to increase to a statistically significant level in the 1st month, 3rd month, and 6th month after the procedure. The 2018 study by Wang et al^21^ found that the Qol score of the patients increased after the procedure. Our study’s results are similar to the results of these studies. There are also studies in the literature in which PAE application shows a reduced Qol score^18,25^ or no effect.^17,26^ Our study determined that the patients’ Qol scores increased to a statistically significant level after the PAE procedure. The patients were followed up, as they had a catheter before the PAE procedure, and the catheter was removed after the procedure. We think that patients’ ability to urinate easily without being dependent on the catheter increases their quality of life.

## Limitations of the study

The fact that the study was conducted only in a single center; the absence of a control group; and the limited number of samples are the limitations of this research.

In conclusion, when we look at the post-procedural results of PAE, we see that there was a reduction in the prostate volume, that the shrinkage was completed in the 6th month, and that the shrinkage did not continue thereafter. The average PSA value decreased in the 1st month, then the rate of decrease continued until the 6th month, after which there was no decrease. It was observed that PAE increased the Qmax value, but continued without significant difference afterwards, and that the IPSS value significantly decreased in the first 3 months. It was observed that there was no significant change in the mean IIEF value. A significant increase was observed in Qol values. In light of all these results, it has been determined that the clinical results of prostatic artery embolization performed with minimally invasive techniques under local anesthesia are quite successful for patients with benign prostatic hyperplasia for whom anesthesia is contraindicated due to advanced age and comorbidities. The procedure is easily tolerated by the patients, and there are no significant complications. We recommend prostatic artery embolization as a safe and effective treatment in this patient group and suggest that studies with larger samples will contribute significantly to the literature.

## Figures and Tables

**Table 1.** Sociodemographic and Clinical Data of the Patients

Features (n = 30)	Mean ± SD (Range)
**Age (years)**	79.3 ± 6.8 (65-89)
**BMI (kg/m²)**	25.3 ± 4.3 (23.1-25.4)
**Charlson comorbidity index**	3.0 ± 1.8 (2.0-4.0)
**PV (cm** **3** **)**	68.0 ± 23.25 (45.00-99.75)
**PSA (ng/mL)**	4.9 ± 1.2 (3.7-6.3)
**Q** **max** **(mL/sec)**	0
**IPSS (point)**	35 ± 3.0 (32-35)
**IEFF (point)**	8.25 ± 0.67 (7.98-8.73)
**Qol (score)**	3.2 ± 0.28 (2.1-3.5)

BMI, body mass index; PV, prostate volume; PSA, prostate-specific antigen; Q_max_, uroflowmetric maximum flow rate; IPSS, international prostatic symptom score; IEFF, international index of erectile function; Qol, quality of life index; SD, standard deviation.

**Table 2.** Clinical Values of the Patients Before and After the Procedure

Clinical Features (n = 30)	**Before the Procedure (**Mean ± SD)	**First Month After the Procedure (**Mean ± SD)	**Third Month After the Procedure (**Mean ± SD)	**Sixth Month After the Procedure (**Mean ± SD)	**Twelfth Month After the Procedure (**Mean ± SD)	*P*
**Prostate volume (cm³)**	68 ± 23.25	61.5 ± 25.5	48.2 ± 15.7	45.5 ± 16.2	45.0 ± 16.0	.001
**PSA (ng/mL)**	4.9 ± 1.2	3.8 ± 1.25	3.5 ± 1.1	2.85 ± 1.05	2.8 ± 1.2	.008
**Qmax (mL/sec)**	0	12 ± 2.0	14 ± 2.5	14 ± 2.0	12.0 ± 3.0	.001
**IPSS (point)**	35.0 ± 3.0	22.5 ± 1.5	18.0 ± 2.0	16.0 ± 2.0	16.0 ± 2.0	.001
**IIEF (point)**	8.25 ± 0.67	8.31 ± 0.6	8.39 ± 0.62	8.42 ± 0.65	8.46 ± 0.70	.32
**Qol (score)**	3.2 ± 0.28	3.06 ± 0.26	4.06 ± 0.27	4.96 ± 0.29	5.09 ± 0.29	.027

SD, standard deviation; PSA, prostate-specific antigen; IPSS, international prostatic symptom score; IEFF, international index of erectile function; Qol, quality of life index.
